# A Review on the Relationship between SGLT2 Inhibitors and Cancer

**DOI:** 10.1155/2014/719578

**Published:** 2014-08-31

**Authors:** Hao-Wen Lin, Chin-Hsiao Tseng

**Affiliations:** ^1^Division of Endocrinology and Metabolism, Department of Internal Medicine, National Taiwan University Hospital, Yun-Lin Branch, Yunlin, Taiwan; ^2^Division of Endocrinology and Metabolism, Department of Internal Medicine, National Taiwan University Hospital, No. 7 Chung-Shan South Road, Taipei 10002, Taiwan

## Abstract

Risk of increasing breast and bladder cancer remains a safety issue of SGLT2 (sodium glucose cotransporter type 2) inhibitors, a novel class of antidiabetic agent. We reviewed related papers published before January 29, 2014, through Pubmed search. Dapagliflozin and canagliflozin are the first two approved SGLT2 inhibitors for diabetes therapy. Although preclinical animal toxicology did not suggest a cancer risk of dapagliflozin and overall tumor did not increase, excess numbers of female breast cancer and male bladder cancer were noted in preclinical trials (without statistical significance). This concern of cancer risk hindered its approval by the US FDA in January, 2012. New clinical data suggested that the imbalance of bladder and breast cancer might be due to early diagnosis rather than a real increase of cancer incidence. No increased risk of overall bladder or breast cancer was noted for canagliflozin. Therefore, the imbalance observed with dapagliflozin treatment should not be considered as a class effect of SGLT2 inhibitors and the relationship with cancer for each specific SGLT2 inhibitor should be examined individually. Relationship between SGLT2 inhibition and cancer formation is still inconclusive and studies with larger sample size, longer exposure duration, and different ethnicities are warranted.

## 1. Introduction

The prevalence of type 2 diabetes mellitus is increasing globally with time [1, 2]. According to an epidemiological study with 370 country years and 2.7 million participants, prevalence of diabetes in adults aged 25 years and older was 9.8% in men and 9.2% in women in 2008, leading to an estimated 173 million men and 173 million women with diabetes [1]. Intensive glycemic control was associated with significantly decreased rates of microvascular and neuropathic complications in type 2 diabetic patients [3, 4]. More intensive glycemic control in newly diagnosed type 2 diabetic patients may reduce long-term cardiovascular disease rates [5]. Along with lifestyle modification, medications with different mechanisms may be needed to achieve glycemic goal.

SGLT2 (sodium glucose cotransporter type 2) inhibitor is a novel class of antidiabetic agent acting independent of effects on insulin resistance or insulin insufficiency. Glucose filtered through the kidneys is reabsorbed into the blood in the proximal tubules via SGLT1 and SGLT2 [6]. SGLT2 accounts for 90% and SGLT1 accounts for 10% of the glucose reabsorbed from the kidneys [7, 8]. SGLT2 is expressed mainly in the kidney cortex [9]. Beside the kidney, SGLT1 is mainly expressed in small intestine and also in trachea, heart, and plasma membranes [6, 9]. Blocking these transporters in the kidney inhibits renal glucose reabsorption and may lower blood glucose levels in diabetic patients. However, nonselective inhibition of SGLT1 and SGLT2 can cause severe diarrhea [10, 11]. Oral agents with high selectivity of SGLT2 have been developed to overcome the shortcoming [11]. Use of selective SGLT2 inhibitors significantly lowers HbA1c levels by 0.5–1.5% without hypoglycemia [12–17]. Use of SGLT inhibitors also showed significant weight loss and reduction of systemic blood pressure [13, 14, 16–18]. Increased genital infection and possibly increased urinary tract infection were noticed but rarely caused drug discontinuation [7, 11, 16, 17, 19].

Currently, more than ten SGLT2 inhibitors are under development [17, 19]. SGLT2 inhibitors under phase II and III clinical trials are listed in Table 1. Dapagliflozin was the first SGLT2 inhibitor approved in the world. It was approved in Europe in November 2012 and in USA in January 2014. Dapagliflozin should be given at an initial dose of 5 mg per day and may be increased to 10 mg per day and it is not recommended in patients with limited renal function (eGFR < 60 mL/minute/1.73 m^2^) [20]. Canagliflozin was approved in Europe and in USA in 2013. It should be given at an initial dose of 100 mg per day and may be increased to 300 mg per day in a once daily dose. Canagliflozin was not recommended in patients with severe renal impairment and end stage renal disease [14].

Patients with diabetes are at an increased risk of bladder cancer compared with those without diabetes [21–26]. Use of some antidiabetic agents (e.g., pioglitazone) might be associated with increased risk of bladder cancer [27–33] and some (e.g., metformin) were associated with lower risk [34]. An imbalance of male bladder cancer and female breast cancer was observed in the treatment arms of dapagliflozin [35]. This ever hindered the approval of dapagliflozin by the FDA in January, 2012. Therefore, it is important to clarify the cancer risk associated with the use of SGLT2.

Publications before January 29, 2014, were searched in the Pubmed using key words including “SGLT1 and cancer,” “SGLT2 and cancer,” “canagliflozin and cancer,” “dapagliflozin and cancer,” “empagliflozin and cancer,” “ipragliflozin and cancer,” “tofogliflozin and cancer,” “luseogliflozin and cancer,” and “BI 44847 and cancer.” Except for the two approved SGLT2 inhibitors (i.e., dapagliflozin and canagliflozin), the incidence of cancer for the other SGLT2 inhibitors was not available. Hence, the present review will focus on the cancer risk associated with dapagliflozin and canagliflozin.

## 2. Dapagliflozin

Among phase 2b and phase 3 trials presented to FDA in 2011, event rates for male bladder cancer and female breast cancer in the active treatment arms of dapagliflozin exceeded the rates expected in an age-matched reference of diabetic population [35]. However, the imbalance was not statistically significant.

More updated data until November, 2013, were gathered through 21 phase 2b and phase 3 trials [20]. The incidence rate ratios of malignancy by tumor type compared to dapagliflozin and nondapagliflozin groups were listed in Table 2. There was no overall imbalance of malignancies [20]. Though not significant, some tumor types (such as bladder cancer and female breast cancer) were more common while others (such as renal tract cancer) were less common in the dapagliflozin group.

### 2.1. Bladder Cancer

At the time of the FDA advisory committee meeting in 2011, 9 cases of bladder cancer were reported on dapagliflozin out of 5,478 patients and one was reported on control out of 3,156 patients among 14 phase 2b and 3 clinical trials [35].

Updated data presented in November, 2013, were gathered from 21 phase 2b and 3 clinical trials of dapagliflozin. With 2000 additional patient-years of exposure compared to those in 2011, no additional case of bladder cancer was identified. All previously detected cases of bladder cancer were males. Subsequent follow-up after the integrated database was locked, a new case of bladder cancer was reported in a female patient who participated in an ongoing add-on sulfonylurea and metformin study [20]. This additional female patient of bladder cancer was a smoker who had hematuria at baseline, and the bladder cancer was diagnosed only 3.5 months after initiation of treatment [20].

Till November, 2013, a total of 10 cases of bladder cancer out of 6,045 patients (0.17%) were noted in the dapagliflozin treatment group compared to 1 case of bladder cancer out of 3,512 patients (0.03%) in the placebo arms [20]. All 10 cases of bladder cancer were reported within 2 years of starting dapagliflozin. All but one showed hematuria, which was the first clinical sign of bladder cancer, within 6 months of starting treatment [20]. The incidence rate remained stable over the first 2 years of drug exposure then fell, with no additional case detected between 2–4 years of exposure. The characteristics of bladder cancer differed from low grade to high grade and from noninvasive to widely metastatic [20]. The biological heterogeneity argued against a single triggering cause for the cancer [20].

A recently published study by Reilly et al. disclosed that, in mice and rats, exposure to dapagliflozin for up to 2 years at greater than a 100-fold human clinical exposure did not increase tumor incidence or urinary bladder proliferative/preneoplastic lesions [36]. In their study, dapagliflozin and its primary metabolite did not affect* in vitro* transitional cell carcinoma (TCC) proliferation rates and dapagliflozin did not enhance tumor growth in nude mice heterotopically implanted with human bladder TCC cell lines [36].

Therefore, the clinical observation of imbalance in bladder cancer associated with dapagliflozin was not supported by animal or* in vitro* studies, and the early diagnosis of bladder cancer might be due to detection bias rather than true causal relationship.

### 2.2. Breast Cancer

In an animal carcinogenicity study, dapagliflozin was administered to Sprague-Dawley rats up to 186-fold human exposure for 90 weeks in males and 105 weeks in females [36]. No mammary tumor was noted in the male rats during study. In the female rats, the most common causes of death in treatment and control groups were benign and malignant mammary and pituitary tumors. However, there was no dapagliflozin related increase in the incidence of these tumors and there was no difference in the time to onset of the tumors [36].

In the data presented in the FDA advisory committee meeting in 2011, 9 cases of breast cancer out of 2,223 female patients (0.4%) were noted in the dapagliflozin treatment group compared to 1 case out of 1,053 female patients (0.09%) in the placebo group [35, 37].

Till November, 2013, the total number of breast cancer cases on dapagliflozin was 12 and was 3 for the control group [20]. Exposure adjusted incidence rate was 0.40 per 100 patient-years (95% CI = 0.21, 0.70) on dapagliflozin and 0.19 (95% CI = 0.04, 0.56) in the control group [20]. The incidence rate ratio decreased from 4.41 (95% CI = 0.57, 200.86) as estimated in 2011 to 2.47 (95% CI = 0.64, 14.10) in 2013 when more patient exposure was included [20].

All female breast cancers were diagnosed within the first year of treatment and were heterogeneous in patient age, tumor type, stage, progesterone/estrogen receptor status, and HER2/neu status [20]. These may not support a causative role for dapagliflozin.

## 3. Canagliflozin

In an animal study, lifetime exposure to canagliflozin in CD-1 mice at doses up to a 14-fold human clinical exposure did not increase the incidence of neoplasms or preneoplastic histological lesions [14].

Canagliflozin was associated with increased neoplasms of renal tubules, adrenals, and testicular Leydig cells (LCT) in Sprague-Dawley rats, which were preceded by carbohydrate malabsorption and disrupted calcium homeostasis [14, 38, 39]. However, canagliflozin does not result in significant carbohydrate malabsorption or calcium imbalance in humans [14].

Due to the increased renal, adrenal, and LCT tumor associated with canagliflozin in rat studies and the clinical imbalance of bladder and breast cancer associated with dapagliflozin, the incidence of these five cancers was followed through 8 phase 3 trials with canagliflozin. There were 6,645 patients who underwent canagliflozin and 3,640 patients who underwent noncanagliflozin treatment.

Till November 15, 2012, there were no reported cases of pheochromocytoma or malignant adrenal tumors in the treatment group [14]. One case of testicular cancer was reported 2 months after starting canagliflozin 100 mg. The patient had an enlarged scrotum a year prior to trial entry and had scrotal pain before trial entry. The prior scrotal abnormality and the short latency period did not support a drug-related causality [14].

The overall incidence of bladder, breast, and renal cancers did not increase in canagliflozin treatment groups while being compared to the noncanagliflozin groups (Table 3) [14, 37].

## 4. Discussion

### 4.1. Class Effect of Cancer Risk Unlikely for SGLT2 Inhibitors

Statistical data did not reveal an overall imbalance of cancer in both canagliflozin and dapagliflozin [14, 20, 35]. An increased incidence of bladder cancer without statistical significance was observed in patients treated with dapagliflozin. However, this was not similarly observed in patients treated with canagliflozin, which actually showed a trend of decreased risk (Table 3). Therefore, the risk of bladder cancer associated with SGLT2 inhibitors is not conclusive and such a risk is unlikely a class effect.

### 4.2. Cancer Risk Not Increased in SGLT2 Knockout Animals

Reilly et al. studied the potential carcinogenic risk of inhibiting SGLT2 in SGLT2^−/−^ mice [36]. The 15-month study included 36 (23 male and 13 female) SGLT knockout mice and 33 (16 male, 17 female) wild type mice. At 15 months, 86% of knockout mice and 85% wild type mice survived. Knockout mice exhibited substantial glucosuria. Microscopic evaluation of urinary bladder, kidneys, liver, heart, pancreas, adrenal glands, thyroids, spleen, female reproductive tract, male sex glands, skin, brain, and skull did not reveal adverse effect of SGLT2 gene deletion. Neither hyperplasia nor neoplasia was observed in the urinary bladder mucosa, urogenital tract, or kidneys of SGLT2 knockout mice [36].

### 4.3. Cancer Cells May Overexpress SGLT

In humans, SGLT1 is overexpressed in many cancers [19, 40]. Inhibition of SGLT1 sensitizes prostate cancer cells to treatment with EGFR (epidermal growth factor receptor) tyrosine kinase inhibitor [40]. High SGLT1 level combined with high MAP17 (membrane-associated protein 17) is a marker for good prognosis in patients with cervical cancer after chemotherapy and radiotherapy [41]. High SGLT1 expression in pancreatic adenocarcinomas was significantly correlated with disease free survival, especially in younger patients [42]. On the other side, overexpression of SGLT1 is related to tumor development and poor prognosis of ovarian carcinoma [43]. In oral squamous cell carcinoma, SGLT1/EGFR expression was inversely related to tumor differentiation [44]. SGLT1/EGFR overexpression in colorectal cancer was related to higher clinical stages though SGLT1 expression was not associated with the prognosis [45].

In a study of lung cancer, there were no significant differences in the level of SGLT1 or SGLT2 gene expression between the primary lung cancers and the normal lung tissues [46]. However, higher SGLT2 expression was found in metastatic lesions of lung cancer compared to primary tumor [46].

Studies above imply that SGLT, especially SGLT1, plays a role in glucose uptake in many cancers. From this point of view, inhibition of SGLT1 and SGLT2 might even be protective in certain cancer types. If there was any positive link between dapagliflozin and bladder cancer, mechanisms other than inhibition of SGLT2 should be considered. Again, relationship between different SGLT2 inhibitors and bladder cancer should be examined individually.

### 4.4. Detection Bias and Increased Bladder Cancer Risk Induced by Glucosuria and Urinary Tract Infection Related to SGLT2 Use

SGLT2 inhibitors block renal glucose reabsorption resulting in increased glucosuria. Increased urinary tract and genital infections were observed in patients treated with SGLT2 inhibitors [7, 8, 15–17, 19]. The increased use of urinalysis in these patients may lead to a detection bias for bladder cancer. However, the possibility of an increased risk of bladder cancer induced by chronic glucosuria and urinary tract infection among patients who use SGLT2 inhibitors cannot be excluded.

In SGLT2^−/−^ mice, lifelong glucosuria did not increase the incidence of bladder tumor [36]. However, patients with diabetes are indeed at an increased risk of bladder cancer compared with those without diabetes [21–26]. Recurrent or chronic urinary tract infection may cause chronic irritation to bladder epithelium and is a potential risk factor of bladder cancer in humans [47–50]. The duration of current human studies on SGLT2 inhibitors is probably not long enough to answer the question and careful postmarketing surveillance should be conducted.

### 4.5. Ethnicity and Sexual Differences

Ethnicity should be taken into consideration while evaluating the risk of bladder cancer. Diabetes associated bladder cancer risk was obviously higher in Asia populations [21]. A population-based matched case—control study performed in the USA, suggested a reduced risk of bladder cancer in women, but not in men, with a history of urinary tract infection [51]. However, a large population-based study in Taiwan disclosed that urinary tract infection is significantly associated with increased bladder cancer risk in both sexes [25].

In phase 2b and phase 3 studies of dapagliflozin presented to the FDA, Asian ethnicities account for less than 20% of the study population [20]. The sample size of the studied Asian population was probably too small to evaluate the effect in this specific ethnic group of patients.

## 5. Conclusions

Molecular evidences and animal studies do not suggest a positive link between exposure to SGLT2 inhibitors and cancer risk. The imbalance of bladder cancer and breast cancer with dapagliflozin treatment in humans could be a result of early diagnosis of preexisting cancer. However, long-term effects should be examined carefully by including larger sample size, with longer exposure duration, and in different ethnicities.

## Figures and Tables

**Table 1:** SGLT2 inhibitors under phase II and phase III clinical trials.

Drug	Company	Phase of clinical trial
Dapagliflozin (BMS-512148)	Bristol-Myers Squibb/AstraZeneca	Phase III (approved in Australia (Oct 2012), Europe (Nov 2012), Mexico (Mar 2013), New Zealand (June 2013), Brazil (July 2013), Argentina (Sep 2013), US (Jan 2014), and Japan (Mar 2014))

Canagliflozin (TA-7284, JAJ-28431754)	Johnson and Johnson and Mitsubishi Tanabe Pharma	Phase III (approved in US (Mar 2013) and Europe (Nov 2013))

Empagliflozin (BI-10773)	Boehringer Ingelheim/Lilly	Phase III (approved in Europe (May 2014))

Ipragliflozin (ASP-1941)	Astellas Pharma and Kotobuki Pharmaceutical Company	Phase III (approved in Japan (Jan 2014)

Tofogliflozin (CSG452)	Roche/Chugai	Phase III

Luseogliflozin (TS-071)	Taisho	Phase III

BI 44847	Boehringer Ingelheim	Phase III

LX4211	Lexicon Pharmaceutical	Phase II

PF-04971729	Pfizer	Phase II

EGT0001442	Theracos	Phase II

GW 869682	GlaxoSmithKline	Phase II

From [17, 19].

**Table 2:** Incidence rate ratio of malignancy by tumor type comparing dapagliflozin versus nondapagliflozin in 21 phase 2b and phase 3 clinical trials (dapagliflozin *N* = 5936 and control *N* = 3403).

Tumor origin	Patients with events	Incidence rate ratio	95% confidence interval
Overall	140	1.03	0.71, 1.51
Bladder	10	5.17	0.68, 233.55
Breast (female only)	15	2.47	0.64, 14.10
Pancreas	8	1.84	0.31, 19.46
Prostate (male only)	17	1.50	0.53, 5.35
Hepatobiliary	3	0.92	0.04, 61.49
Thyroid and endocrine	10	0.88	0.19, 4.46
Skin	31	0.83	0.37, 1.91
Respiratory and mediastinal	15	0.79	0.24, 2.81
Female reproductive (female only)	4	0.74	0.05, 10.74
Metastases and site unspecified	5	0.56	0.07, 8.96
Gastrointestinal	10	0.51	0.13, 3.19
Renal tract	5	0.40	0.03, 3.82
Blood/lymphatic	7	0.37	0.05, 2.35
Musculoskeletal and soft tissue	1	*∞*	0.010, *∞*

Source: reference [20, 35]: data obtained from 21 phase 2b and 3 clinical trials, data cutoff date: Nov 4, 2013; a new case of bladder cancer found in an ongoing add-on to sulfonylurea and metformin phase 3 trial is not included. The incidence rate ratio with 95% CI including the case is 6.11 (0.827–272.00).

**Table 3:** Incidence rates of bladder and breast cancer with canagliflozin exposure.

Canagliflozin	*N*	Subjects with events (%)	Rate per 1000 patient-year
Bladder Cancer			
Canagliflozin 100 mg	3139	2 (0.06)	0.44
Canagliflozin 300 mg	3506	3 (0.09)	0.63
All canagliflozin	6645	5 (0.07)	
All noncanagliflozin	3640	4 (0.11)	0.84

Breast Cancer			
Canagliflozin 100 mg	1313	5 (0.38)	2.61
Canagliflozin 300 mg	1514	7 (0.46)	3.39
All canagliflozin	2827	12 (0.42)	
All noncanagliflozin	1501	6 (0.4)	3.05

Source: reference [14], data obtained from 8 phase 3 clinical trials, data cutoff date: Nov 15, 2012.
